# Comparison of Preoperative and Postoperative Conventional and Speckle Tracking Echocardiographic Parameters in Living Liver Donors

**DOI:** 10.7759/cureus.75998

**Published:** 2024-12-19

**Authors:** Gulsum Bingol, Fulya Avci Demir, Özge Özden, Kardelen Ohtaroglu Tokdil, Serkan Unlu, Muharrem Nasıfov, Hızır Okuyan, Ferit Boyuk, Ismail P Canbolat, Volkan Camkiran, İbrahim Sarı, Barıs Okcun, Ahmet Kargı, Kamil Yalcın Polat

**Affiliations:** 1 Cardiology, Arel University Medical Faculty, Istanbul, TUR; 2 Cardiology, Memorial Bahcelievler Hospital, Istanbul, TUR; 3 Cardiology, Antalya Medical Park Hospital, Antalya, TUR; 4 Cardiology, Memorial Bahçelievler Hospital, Istanbul, TUR; 5 Cardiology, Kanuni Sultan Süleyman Training and Research Hospital, Istanbul, TUR; 6 Cardiology, Gazi University Faculty of Medicine, Ankara, TUR; 7 Cardiology, Liv Bona Dea Hospital, Baku, AZE; 8 Cardiology, Konya Beyhekim Training and Research Hospital, Konya, TUR; 9 Cardiology, Health Sciences University, Yedikule Chest Diseases and Thoracic Surgery Training and Research Hospital, Istanbul, TUR; 10 Cardiology, Bahcesehir University Medical Park Goztepe Hospital, Istanbul, TUR; 11 Transplant, Memorial Bahcelievler Hospital, Istanbul, TUR

**Keywords:** echocardiography, hepatectomy, left atrial strain, living donor liver transplant (ldlt), myocardial strain imaging

## Abstract

Introduction

We aimed to assess whether partial hepatectomy has an influence on conventional and speckle tracking parameters on echocardiography in living liver donors in the early postoperative period.

Methods

This study was a retrospective study to investigate the cardiac effects of liver donation after the transplant operation in a high-volume liver transplant center. Ninety living liver donors were included in the study. The echocardiographic images were obtained from 90 living liver donors before and five to seven days after the operation. The echocardiographic examinations were evaluated with a Philips Epiq 7 ultrasound system (Philips Ultrasound; Bothell, WA, USA) by experienced cardiologists in accordance with the recommendations of the American Society of Echocardiography. These included M-mode, two-dimensional imaging, tissue Doppler assessment at the septal and lateral mitral annulus, and strain imaging in all patients at rest in the left decubitus position. The changes in echocardiographic parameters in living liver donors were analyzed. Paired T-test was used to assess significant differences.

Results

The left ventricular (LV) global longitudinal strain (GLS), right ventricular (RV) GLS, and RV free wall LS, reservoir phase of left atrial strain (LAS-r), conduit phase of LAS (LAS-cd) did not show significant changes after the operation (for all, p>0.05). However, the absolute atrial contraction phase of LAS (LAS-ct) mean value increased significantly (14.2±8.8 vs 16.6±8.3, p=0.025) postoperatively. Among the conventional echo parameters, isovolumic relaxation time (IVRT) and the E/A ratio demonstrated notable postoperative alterations. The mean IVRT (87.3 ± 22.4 vs. 80.8 ± 18.1, p=0.014) and E/A ratio (1.5 ± 0.5 vs. 1.3 ± 0.3, p=0.012) exhibited a postoperative decline.

Conclusions

To the best of our knowledge, our study is the first to evaluate the effect of partial hepatectomy on cardiac functions by echocardiography in living liver donors, and there was no deterioration in the functions of both the ventricles and left atrium.

## Introduction

Liver transplantation (liver Tx) has become the only lifesaving and decisive treatment option for end-stage liver diseases, acute liver failure, various metabolic diseases, and several liver tumors. The liver donor candidate can be a cadaver or a living volunteer. However, liver Tx from a cadaver fails to provide a necessary number of transplantable organs. Therefore, liver Tx from a living donor has started to be the preferred option, with increasing frequency in some regions, in order to deliver the optimal treatment before the development of liver failure [1].

Worldwide, approximately 32,000 liver Tx are performed annually [2]. Although living donation was introduced in 1996, 8421 living donors (LD) were reported in 14,6782 liver Tx in Europe. Recent data from 2020 shows that 17.8% of liver Tx are performed from a living donor [3]. 

LD liver Tx has a low risk of donor death. In addition, possible perioperative cardiac impairment has not been elucidated in the literature. The mortality rate stands around 0.2% and is mainly related to sepsis, liver failure, cerebral hemorrhage, and pulmonary embolism [4]. Nevertheless, evaluation of cardiac function is critical to assess perioperative risk and complications [5]. Conventional assessment includes systolic and diastolic functions of both ventricles.

Along with the conventional assessment, strain imaging can detect subclinical impairment of cardiac functions as a reproducible, valuable, prognostic tool [6,7]. Global longitudinal strain (GLS) has become a clinically feasible, guideline-recommended parameter in addition to left ventricular (LV) ejection fraction (LVEF) for the evaluation of LV function [8]. Over the last few years, right ventricular (RV) and left atrium (LA) strain imaging has emerged as new tools superior to conventional echocardiographic evaluation [9]. In particular, the LA strain has been recommended in addition to the assessment of LV diastolic function as a very strong parameter of diastolic dysfunction and a robust predictor of heart failure, atrial fibrillation, or cardiovascular death [10,11].

We investigated the effects of partial hepatectomy on cardiac function by comparing preoperative and postoperative conventional parameters and LV, LA, and RV strain values in patients who underwent partial hepatectomy as organ donors.

## Materials and methods

This retrospective study investigated the cardiac effects of liver donation after the transplant operation in a high-volume liver transplant center. An experienced liver-heart team has been established to evaluate the patients pre- and postoperatively and closely follow their cardiac status in our hospital.

The study protocol was approved by the local ethics committee of our institution (date: 23.02.2021/ number:11). Patients over 18 years old with no other cardiac diseases and comorbidities with good quality echocardiographic images for strain analysis were included. Patients with inadequate image quality on echocardiography were excluded from the study. All patients underwent the transplant operation by the same transplant team at our hospital between June 2020 and February 2021.

Echocardiographic evaluation 

The transthoracic echocardiography (TTE) examinations were analyzed according to the recommendations of the American Society of Echocardiography, including M-mode, two-dimensional imaging, tissue Doppler assessment at the septal and lateral mitral annulus and strain imaging in all patients at rest in the left decubitus position [12]. Routine cardiac assessment includes pre- and postoperative echocardiographic evaluation of the donors and the transplant recipients. Postoperative echocardiography is performed five to seven days after the operation before being routinely discharged. The echocardiographic examinations were evaluated with a Philips Epiq 7C (Philips Ultrasound; Bothell, WA, USA) and broadband multifrequency transducers by experienced cardiologists. Patients with suboptimal imaging quality were excluded from the study. Two independent cardiologists analyzed the echocardiographic images, who are particularly interested and trained in cardiovascular imaging and blinded to the other measurements, using a commercially available workstation (QLAB version 13).

Data analysis

Speckle tracking echocardiography (STE) was performed on four consecutive cycles of two-dimensional LV images from the three standard apical views for LV and LA; RV-focused apical four-chamber with a mean frame rate between 71 and 85 frames/second according to the latest guidelines [13]. Images were considered adequate for strain analysis if endocardial and epicardial borders were clearly distinguishable, all cardiac segments were included in the image area, and good tracking was available. For LA strain analysis, LA images were considered adequate if they included the entire LA roof in both apical four-chamber and two-chamber views. A novel 2D strain analytical software (AutoStrain, Philips) that defines the region for automatically tracking around a line and allows changes manually after that was used for strain analysis. Tracking quality was visually checked by comparing the endocardial line's motion with the underlying myocardium's motion by the investigator. Importantly, if regional tracking was suboptimal in more than two myocardial segments in a single image, the GLS should not be calculated. In the segments with poor tracking, the border was readjusted manually until the best possible tracking was achieved. Furthermore, the RV GLS and RV free wall strain (RV-FWSL), components of LAS (atrial reservoir (LAS-r), conduit (LAS-cd), and contractile (LAS-ct) function) were examined by following recommendations of the European Society of Cardiology [13]. The application of the same 2D STE technique to strain analysis of different ventricles is controversial because the shape, structure, and function of the LA and RV are different from those of the LV. This can be the advantage of the AutoStrain method over the conventional method of manual strain analysis.

Statistical analysis

The statistical analyses presented continuous variables as mean ± standard deviation. Categorical data was given as frequencies or percentages, and continuous variables were examined by the Kolmogorov-Smirnov test to check for distribution normality. Paired t-test and Wilcoxon were utilized to compare parametric and nonparametric continuous variables before and after operation, respectively. A two-tailed p-value of <0.05 was considered statistically significant. All data were assessed using Statistical Package for the Social Sciences (version 24; IBM Inc., Armonk, NY, USA).

## Results

The study included 90 living liver donors (39 female, 43.3%, and 51 male, 56.7%) who underwent a liver donation operation. The average age of the donors was 35.6 ± 9.7 years. The basal clinical data of the patients are presented in Table 1. 

**Table 1:** Clinical and biochemical features of living donors BMI - body mass index; GFR - glomerular filtration rate; ALT - alanine aminotransferase; AST - aspartate aminotransferase; LDH - lactate dehydrogenase; Hct - hematocrit; Hb - hemoglobin; PLT - platelet; Na - sodium; K - potassium; LDL - low-density lipoprotein; HDL - high-density lipoprotein; Fe+2 - ferrous

Variables	Mean (SD)
Age (y)	35.6 ± 9.7
BMI (kg/m²)	25.4 ± 3.6
Creatinine (mg/dL)	0.81 ± 0.2
ALT U/L)	19.3 ± 18.3
AST U/L)	22 ± 27.8
LDH U/L)	197.3 ± 94.4
Glucose (mg/dL)	96 ± 19.5
FT4 (ng/dL)	1.3 ± 0.3
TSH (mIU/L)	2.1 ± 1.2
Hct (%)	43.3 ± 4.6
Hb (g/dL)	14.3 ± 1.8
Plt (10^3^/dL)	256.5 ± 58.9
GFR (mL/sec/1,7)	107.8 ± 20
Na (mmol/L)	139.3 ± 2.2
K (mmol/L)	4.3 ± 0.3
LDL (mg/dL)	104.1 ± 33.4
HDL (mg/dL)	48 ± 13.2
Total Cholestrole (mg/dL)	176.5 ± 38.3
Triglyceride (mg/dL)	124.8 ± 73.7
Fe^++ (^mg/dL)	90.9 ± 42.7
Ferittin (mg/L)	89.7 ± 71.1

Results of routine echocardiographic parameter alterations after partial hepatectomy in living liver donors

**Figure 1:**
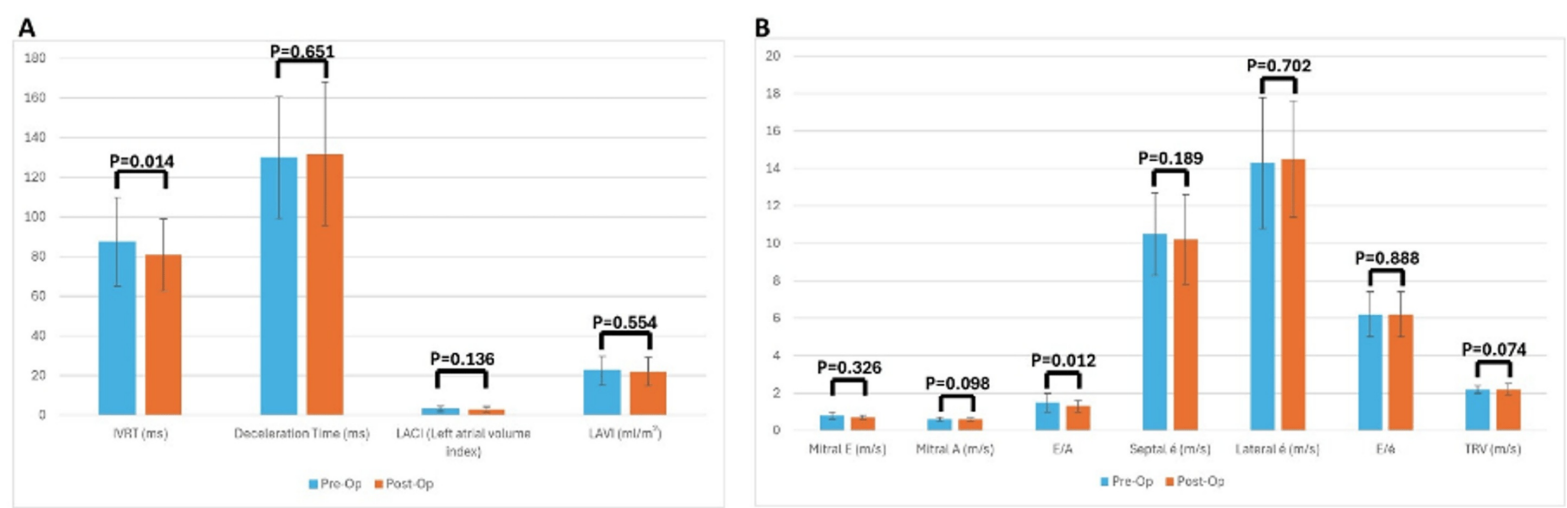
Pre- and postoperative changes in echocardiographic parameters

Deceleration time, mitral E, mitral A, septal é, lateral é, E/é, tricuspid regurgitation velocity (TRV), left atrioventricular coupling index (LACI), left atrial volume index (LAVI), left ventricular end-diastolic volume (LVEDV) and TAPSE averages did not show a significant change after the operation (p>0.05; Table 2). However, isovolumic relaxation time (IVRT) E/A ratios showed significant changes post-operatively. As seen in Table 2, the mean of IVRT (87.3 ± 22.4 vs. 80.8 ± 18.1, p=0.014) and mean of E/A ratio (1.5 ± 0.5 vs. 1.3 ± 0.3, p=0.012) decreased after the operation (Figure 1).

**Table 2:** Alteration in diastolic functions according to partial hepatectomy IVRT - isovolumetric relaxation time; TRV - tricuspid regurgitation velocity; LAVI - left atrial volume index; LACI - left atrial coupling index; LVEDV - left ventricular end-diastolic volume; TAPSE - tricuspid annular plane systolic excursion

Variables	Pre-op (n=90)	Post-op (n=90)	p-value
IVRT (msec)	87.3 ± 22.4	80.8 ± 18.1	0.014
Deceleration time (ms)	129.7 ± 30.7	131.7 ± 36.2	0.651
Mitral E (m/s)	0.8 ± 0.2	0.7 ± 0.1	0.326
Mitral A (m/s)	0.6 ± 0.1	0.6 ± 0.1	0.098
E/A	1.5 ± 0.5	1.3 ± 0.3	0.012
Septal é (m/s)	10.5 ± 2.2	10.2 ± 2.4	0.189
Lateral é (m/s)	14.3 ± 3.5	14.5 ± 3.1	0.702
E/é	6.2 ± 1.2	6.2 ± 1.2	0.888
TRV (m/s)	2.2 ± 0.2	2.2 ± 0.3	0.074
LACI (Left atrial volume index/ TDI- a’)	5.2 ± 16.3	2.9 ± 1.5	0.196
LAVI (ml/m^2^)	22.4 ± 7.3	21.9 ± 7.1	0.554
LVEDV (ml)	77.1 ± 19.4	75 ± 16.3	0.217
TAPSE (cm)	2.3 ± 0.4	2.6 ± 2.2	0.142

Results of STE changes after partial hepatectomy in living liver donors

The mean of RV-FWSL, RV-GLS, LV-GLS, LAS-r, and LAS-cd did not change significantly after the operation (p>0.05). In addition, as seen in Table 3, it was found that the LAS-ct showed a significant change (p=0.025).

**Table 3:** Alterations in speckle tracking echocardiography components according to liver transplantation RV-FWSL - right ventricular free wall longitudinal strain; RV-GLS - right ventricular global longitudinal strain; LV-GLS - left ventricular global longitudinal strain; LAS-r - left atrium reservoir strain; LAS-cd - left atrium conduit strain; LAS-ct - left atrium contractile strain

Variables	Pre-op (n=90)	Post-op (n=90)	p-value
RV-FWSL	-28.5 ± 5.7	-28.7 ± 5	0.688
RV-GLS	-24.8 ± 5.1	-25.3 ± 4.9	0.285
LV-GLS	-20.8 ± 4.2	-21.4 ± 3.3	0.194
LAS-r	49.4 ± 15.4	50 ± 16.6	0.766
LAS-cd	-35.1 ± 12.2	-33.6 ± 16.6	0.397
LAS-ct	-14.2 ± 8.8	-16.6 ± 8.3	0.025

## Discussion

In this study, we compared pre- and postoperative cardiac functions using STE and conventional echocardiography parameters in living liver donors who underwent partial hepatectomy.

The findings of our study can be summarized as follows: 1) the mean of RV-FWSL, RV-GLS, LV-GLS, LAS-r, and LAS-cd did not change significantly after the operation, but the LAS (LAS-ct) mean value increased significantly; 2) isovolumic relaxation time (IVRT) and E/A ratios decreased after the operation.

Liver Tx can be performed from deceased or living donors. Cultural and traditional differences may cause a need for a decent number of transplantable organs, and therefore, the recipients encounter the problem of long waiting periods till the liver Tx operation. Thus, LDLT is a lifesaving alternative for patients with end-stage liver disease, and it has similar postoperative results with deceased donor liver transplantation (DD liver Tx) [14,15].

In Europe and North America, the need for a graft is mostly met by the cadaveric organ pool, and LD liver Tx make up a big part of the donor pool in Turkey and most Asian countries. However, LD liver tx has advantages and disadvantages. The major advantages of LD liver Tx are short waiting time, short cold ischemia duration, being an elective surgical procedure, and easy access to liver grafts for patients in need of urgent liver Tx [16,17]. 

Living donors are individuals with no health problems who actually obtain no benefit at the end of the surgical procedure, which may result in mortality and morbidity [1]. Thus, saving and continuing their healthy status is of paramount importance. Despite all that, the obvious benefits to liver recipients are found to be superior compared to the risks of operative morbidity and mortality of donors [18]. 

Although the LD liver Tx rate is increasing, there is no data on the pre- and postoperative cardiac status of donors. To the best of our knowledge, this is the first echocardiographic study using conventional and STE parameters to assess changes in cardiac systolic and diastolic functions following LD liver Tx in a totally healthy group of patients.

In this study, we compared preoperative and postoperative echocardiographic data sets, including strain analysis of living liver donors, to evaluate the effect of partial hepatectomy on cardiac functions in our hospital, which is one of the most experienced centers in our country with a low amount of cadaver donor despite a high amount living donors. The strain method is mainly used in cases where subclinical damage detection is essential, such as oncological patients under chemotherapy. Additionally, STE has been demonstrated as a meticulous quantitative method for LV function, and this tool has been described for evaluating regional and global LA function, too [19,20]. Atrial strain was investigated in several diseases, such as hypertension, diabetes, heart failure, atrial fibrillation, ischemic heart disease, and valvular diseases [21].

Our study observed no significant difference in LV GLS, RV GLS, RV FWLS, LAS-r, and LAS-cd after partial hepatectomy. However, there was a statistically significant increase in postoperative LAS-ct values compared to preoperative LAS-ct values.

It has already been shown that LASct provides information about LA contractility. It also depends on venous return and LV end-diastolic pressure. The LAsct is related to atrial contractility, venous return, and LV end-diastolic pressure [22]. We did not collect any data on the postoperative volume status of our patients. However, we consider that the increased LA-ct values may be related to the volume overload after the operation.

On the other hand, no precise data on normal atrial contractile strain is available and it shows a wide range from 14.0% to 25.0% [23]. In our study, the values in the patients' pre- and postoperative LAS-ct analysis were within normal range. Additionally, no significant changes were observed in the LAS-r and LAS-cd values, which are mainly effective in evaluating LA functions and are used more in clinical practice, especially for diastolic functions. Due to these two conditions, this statistically significant increase was considered clinically insignificant.

Previous studies on the cardiac effects of liver transplantation were only on the recipients. In addition, with this study, we have shown that liver transplantation from a living donor does not have evident and subclinical effects on the heart after partial hepatectomy.

Limitations 

Our study has several limitations. First, it is retrospective in nature, which may introduce selection bias and limit the generalizability of the findings. Second, only donors with high-quality echocardiographic images were included, which may have led to the exclusion of a larger pool of potential participants and could limit the representativeness of the sample. Third, the study focused on early postoperative changes, and therefore, we lack data on the long-term effects of partial hepatectomy on cardiac function. Finally, while we found no significant changes in cardiac function, subtle changes not captured by conventional echocardiography or strain imaging may still exist, and further studies with longer follow-up periods are needed to assess potential long-term effects.

## Conclusions

This study is the first to assess the early effects of partial hepatectomy on cardiac function in living liver donors using conventional and speckle-tracking echocardiography. Our results suggest that partial hepatectomy does not significantly affect ventricular or left atrial function. Although a small increase in left atrial contractility (LAS-ct) was observed, this change is likely due to physiological factors like fluid shifts rather than cardiac dysfunction.

Clinically, these findings suggest that living liver donors do not require intensive cardiac monitoring postoperatively, as their cardiac function remains stable. However, the increase in LAS-ct may prompt clinicians to monitor for potential volume overload. Overall, this study provides valuable insights for optimizing postoperative care and reducing unnecessary interventions in living liver donors.
